# Supplementary material for: OptoRheo: Simultaneous in situ micro-mechanical sensing and imaging of live 3D biological systems
Source: Commun Biol. 2023 Apr 28;6:463. doi: 10.1038/s42003-023-04780-8 (PMC10147656; doi:10.1038/s42003-023-04780-8)
Supplement: Supplementary file 1 — Supplementary Materials [file 42003_2023_4780_MOESM1_ESM.pdf]

SUPPLEMENTARY MATERIALS

OptoRheo: Simultaneous *in situ* micro-mechanical sensing and imaging of live 3D biological systems

Tania Mendonca, Katarzyna Lis-Slimak, Andrew B. Matheson, Matthew G. Smith, Akosua B. Anane-Adjei, Jennifer C. Ashworth, Robert Cavanagh, Lynn Paterson, Paul A. Dalgarno, Cameron Alexander, Manlio Tassieri, Catherine L. R. Merry and Amanda J. Wright

Supplementary Figures

1. Sample preparation

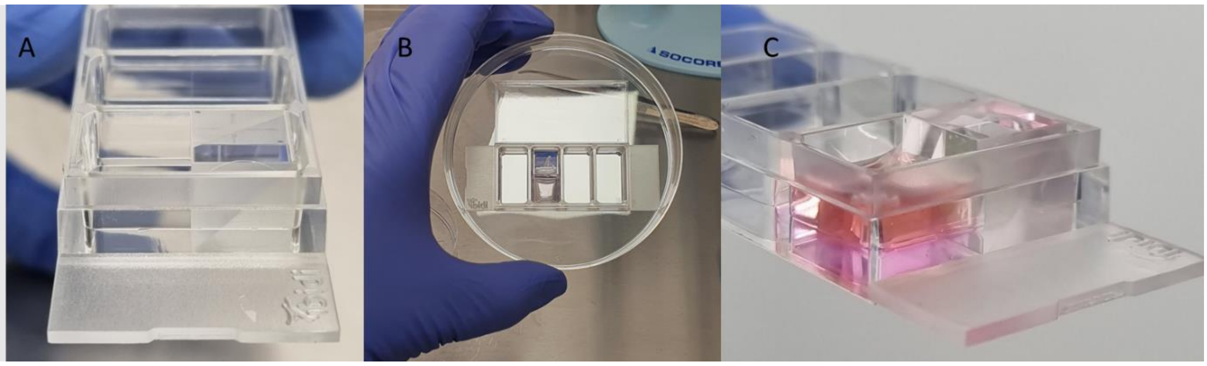

Fig S1: Sample set up: A. Side view of the 4 µ-well chambered coverslip with a 10 mm beam splitter cube inserted with the reflective surface facing the empty half of the chamber. B. Top view of a sample with the gel cast next to the beam splitter cube. C. Side view of the peptide hydrogel topped up with medium next to the beam splitter cube.

2. Light sheet properties

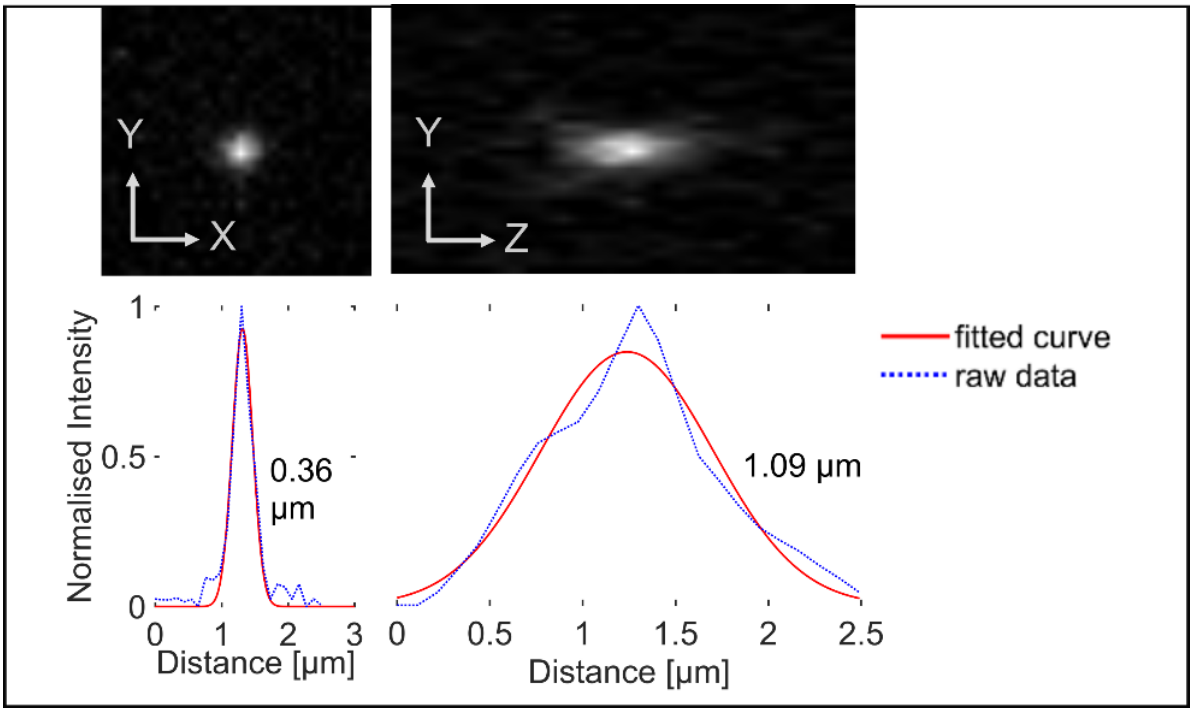

Fig S2: Lateral (left) and axial (right) point spread functions (PSFs) of the system along the XY (0.36 µm FWHM) and YZ (1.09 µm FWHM) planes measured using fluorescent sub-diffraction sized microspheres (diameter = 200 nm, λ_{ex} / λ_{em} = 532 nm / 580 nm) at ~200 µm from the coverslip.

Supplementary video information

SV1: **MDA-MB-231 (tdTomato) cells changing morphology** in 3D within a hydrogel matrix supplemented with collagen I (unlabelled). The video was acquired over ~7 hours with a 10 min time interval between frames. Changes in ECM rheology and cell morphology appear related as a more compliant gel at the start of the video (see Table S1 below) precedes cell elongation while an increase in stiffness (computed as G_0' using Equation 4 from Methods) around 6 hours into the experiment corresponds with a retracted cell morphology.

Time	Measurement location 1 (G_0' [Pa])	Measurement location 2 (G_0' [Pa])	Measurement location 3 (G_0' [Pa])
0 min	2.3×10^{-2}	2.0×10^{-2}	1.5×10^{-2}
120 min	1.5×10^{-2}	0.7×10^{-2}	0.4×10^{-2}
240 min	1.2×10^{-2}	2.2×10^{-2}	3.2×10^{-2}
360 min	20.2×10^{-2}	22.9×10^{-2}	24.0×10^{-2}

Table S1: Microrheology measurements depicted in supplementary video SV1 (clockwise from bottom) over the time course of the experiment.

SV2: **MDA-MB-231 (tdTomato) cells migrating** in 3D within a hydrogel matrix supplemented with collagen I (unlabelled). The video was acquired over 4 hours with a 10 min time interval between frames. Rheology measurements showed a more compliant region (2×10^{-2} Pa) near (~ 50 µm) the migratory path depicted as a dark pink sphere as opposed to farther away (6×10^{-2} Pa at ~80 µm away) depicted as a bright pink sphere.
