# Supplementary material for: OptoRheo: Simultaneous in situ micro-mechanical sensing and imaging of live 3D biological systems
Source: Commun Biol. 2023 Apr 28;6:463. doi: 10.1038/s42003-023-04780-8 (PMC10147656; doi:10.1038/s42003-023-04780-8)
Supplement: Supplementary file 2 — Description of Additional Supplementary Files [file 42003_2023_4780_MOESM2_ESM.pdf]

Description of Additional Supplementary Files

File name: Supplementary Video 1 (SV1) MDA-MB-231 cells changing morphology

Description: Video of MDA-MB-231 (tdTomato) cells changing morphology in 3D within a hydrogel matrix supplemented with collagen I (unlabeled). The video was acquired over ~7 hours with a 10 min time interval between frames. Changes in ECM rheology and cell morphology appear related as a more compliant gel at the start of the video (see Table S1 in supplementary materials) precedes cell elongation while an increase in stiffness (computed as [illegible] using equation 4 from Methods) around 6 hours into the experiment corresponds with a retracted cell morphology.

File name: Supplementary Video 2 (SV2) MDA-MB-231 cells migrating

Description: Video of MDA-MB-231 (tdTomato) cells migrating in 3D within a hydrogel matrix supplemented with collagen I (unlabeled). The video was acquired over 4 hours with a 10 min time interval between frames. Rheology measurements showed a more compliant region (2 x 10-2 Pa) near (~ 50 µm) the migratory path depicted as a dark pink sphere as opposed to farther away (6 x 10-2 Pa at ~80 µm away) depicted as a bright pink sphere.
