# Supplementary material for: OptoRheo: Simultaneous in situ micro-mechanical sensing and imaging of live 3D biological systems
Source: Commun Biol. 2023 Apr 28;6:463. doi: 10.1038/s42003-023-04780-8 (PMC10147656; doi:10.1038/s42003-023-04780-8)
Supplement: Supplementary file 5 — Reporting Summary [file 42003_2023_4780_MOESM5_ESM.pdf]

nature portfolio

Corresponding author(s): Dr Tania Mendonca

Last updated by author(s): Mar 20, 2023

Reporting Summary

Nature Portfolio wishes to improve the reproducibility of the work that we publish. This form provides structure for consistency and transparency in reporting. For further information on Nature Portfolio policies, see our Editorial Policies and the Editorial Policy Checklist.

Statistics

For all statistical analyses, confirm that the following items are present in the figure legend, table legend, main text, or Methods section.

n/a	Confirmed	
☐	☒	The exact sample size (*n*) for each experimental group/condition, given as a discrete number and unit of measurement
☐	☒	A statement on whether measurements were taken from distinct samples or whether the same sample was measured repeatedly
☒	☐	The statistical test(s) used AND whether they are one- or two-sided<br>*Only common tests should be described solely by name; describe more complex techniques in the Methods section.*
☒	☐	A description of all covariates tested
☒	☐	A description of any assumptions or corrections, such as tests of normality and adjustment for multiple comparisons
☐	☒	A full description of the statistical parameters including central tendency (e.g. means) or other basic estimates (e.g. regression coefficient) AND variation (e.g. standard deviation) or associated estimates of uncertainty (e.g. confidence intervals)
☒	☐	For null hypothesis testing, the test statistic (e.g. *F*, *t*, *r*) with confidence intervals, effect sizes, degrees of freedom and *P* value noted<br>*Give P values as exact values whenever suitable.*
☒	☐	For Bayesian analysis, information on the choice of priors and Markov chain Monte Carlo settings
☒	☐	For hierarchical and complex designs, identification of the appropriate level for tests and full reporting of outcomes
☒	☐	Estimates of effect sizes (e.g. Cohen's *d*, Pearson's *r*), indicating how they were calculated

Our web collection on statistics for biologists contains articles on many of the points above.

Software and code

Policy information about availability of computer code

Data collection	Figshare https://doi.org/10.6084/m9.figshare.c.5969601.v1
Data analysis	Data extraction and analysis was performed in MATLAB (2019b; MathWorks, Nattick, MA), Complex moduli were computed using a bespoke MATLAB-based app which can be downloaded here: 10.6084/m9.figshare.19672911

For manuscripts utilizing custom algorithms or software that are central to the research but not yet described in published literature, software must be made available to editors and reviewers. We strongly encourage code deposition in a community repository (e.g. GitHub). See the Nature Portfolio guidelines for submitting code & software for further information.

Data

Policy information about availability of data

All manuscripts must include a data availability statement. This statement should provide the following information, where applicable:

- Accession codes, unique identifiers, or web links for publicly available datasets
- A description of any restrictions on data availability
- For clinical datasets or third party data, please ensure that the statement adheres to our policy

Figure data are available to download from Figshare (https://doi.org/10.6084/m9.figshare.c.5969601.v1)

Human research participants

Policy information about studies involving human research participants and Sex and Gender in Research.

Reporting on sex and gender	N/A
Population characteristics	N/A
Recruitment	N/A
Ethics oversight	N/A

Note that full information on the approval of the study protocol must also be provided in the manuscript.

Field-specific reporting

Please select the one below that is the best fit for your research. If you are not sure, read the appropriate sections before making your selection.

☒ Life sciences ☐ Behavioural & social sciences ☐ Ecological, evolutionary & environmental sciences

For a reference copy of the document with all sections, see nature.com/documents/nr-reporting-summary-flat.pdf

Life sciences study design

All studies must disclose on these points even when the disclosure is negative.

Sample size	MCF-7 cluster study: measurements taken at multiple probes (naked gels = 11; gels with collagen alone = 11; gels with cells alone = 13 and gels with cells and collagen = 14 with repeated measures acquired at each probe on three consecutive days.<br><br>Spheroid study: N = 2 spheroids with repeated measurements taken at probes at fixed distances from the edge of the spheroid (4 µm, n = 4; 6 µm, n = 4; 12 µm, n = 3 and 30 µm, n = 7)
Data exclusions	We confirm that no data were excluded from the analysis
Replication	MCF-7 cluster study: N = 3<br>Spheroid study: N = 2
Randomization	Position of each sample condition in the chambered coverslip was randomised between repeats and the order of measurement for each sample condition was randomised between the three days to control for any potential effect of measurement order.
Blinding	Analysis was automated and batch processing was utilised as much as possible to remove any user bias.

Reporting for specific materials, systems and methods

We require information from authors about some types of materials, experimental systems and methods used in many studies. Here, indicate whether each material, system or method listed is relevant to your study. If you are not sure if a list item applies to your research, read the appropriate section before selecting a response.

Materials & experimental systems

n/a	Involved in the study	
☒	☐	Antibodies
☐	☒	Eukaryotic cell lines
☒	☐	Palaeontology and archaeology
☒	☐	Animals and other organisms
☒	☐	Clinical data
☒	☐	Dual use research of concern

Methods

n/a	Involved in the study	
☒	☐	ChIP-seq
☒	☐	Flow cytometry
☒	☐	MRI-based neuroimaging

Eukaryotic cell lines

Policy information about cell lines and Sex and Gender in Research

Cell line source(s)	tdTomato MCF7, GFP MCF7, tdTomato MBA-MD-231: modified human female breast cancer cell lines were a gift from Prof.

Cell line source(s)	Anna Grabowska's lab, University of Nottingham. The lines were produced by lentiviral transduction of cells originally obtained under MTA from NCI as part of the NCI-60 panel.
Authentication	The cells have come directly from a recognized source (NCI)
Mycoplasma contamination	All cell lines are tested routinely for mycoplasma (montly) none of the lines used in this study tested positive at any point.
Commonly misidentified lines<br>(See ICLAC register)	A search using the register gave no return.
